# Socioeconomic status, perceptions and obesity among adolescents in Jordan

**DOI:** 10.11604/pamj.2019.34.148.19641

**Published:** 2019-11-14

**Authors:** Abdulhakeem Mahmoud Okour, Rami Abdullah Saadeh, Manar Hisham Hijazi, Hana Eyyad Al Khalaileh, Mahmoud Ahmad Alfaqih

**Affiliations:** 1 Jordan University of Science and Technology, Irbid, Jordan

**Keywords:** Obesity, adolescents, perceptions, socioeconomic status, Jordan

## Abstract

**Introduction:**

Prevalence of obesity among adolescents constitutes a serious public health problem. We aimed to investigate the association between socioeconomic factors and obesity perceptions, and prevalence of overweight and obesity among school adolescents.

**Methods:**

A cross-sectional study that included students 12 to 17 years old participating from six schools that were randomly selected in Northern Jordan. Body mass index (BMI) measurements and interview questions were completed by trained researchers. A total of 701 were successfully involved in the analysis.

**Results:**

Students involved were 53.6% males and 46.4% females. Students with normal BMI had a mean BMI of 21.45 (+1.76). Those who were obese, or overweight were 202 (28.8%) students and had a BMI of 29.1 (+4.05). Family monthly income, mother's education and working status among other socioeconomic status factors were significantly associated with children’s overweight or obesity. Perceptions about obesity including meal choices, interest in self body weight, considering someone in family as obese and instructions at school were statistically significant as well.

**Conclusion:**

Family factors, adolescents' pocket money and perceptions about obesity were significant predictors of obesity among adolescents in Jordan. Effective intervention strategies should be implemented in schools and other primary care settings to reduce the relatively high prevalence of adolescent's obesity observed in this study.

## Introduction

At the individual level, childhood obesity is defined as “a result of an imbalance between the calories a child consumes as fat foods and beverages and the calories a child uses to maintain normal growth, metabolism, and physical activities” [1]. Overweight and obesity are increasing dramatically and rapidly in both children and adults. Many health professionals are sounding the alarm about the problems of obesity-related disability and death as well as the socioeconomic burden on health care systems [2]. Studies have reported that the prevalence of obesity has been associated with age [1], gender [3], physical activity [4], dietary patterns [5], and socioeconomic status since families with less monthly household income suffer from an increase in overweight and obesity [6]. Several international and regional studies indicate that factors causing obesity are multi-factorial in nature. These factors may include genetic, behavioral, environmental, cultural, and socioeconomic factors [5-8]. Low socioeconomic status (SES) of individuals or families usually accompanies living in poor settings which offers an environment that allows easy access to unhealthy food and encourages physical inactivity. Families that have a history of low SES often provides information of unhealthy behavior, poor preferences of food selection, shopping, cooking, and exercising [5,7,9,10]. Another important social factor that is related to obesity is individuals' self-image and perceptions about health. Children behave in a certain way to reflect special patterns of thoughts and behaviors that are shared with other groups [11-13]. For this reason, peers have positive and negative influences on the children's behavior. Many children and adolescents spend their time with peers in fast-food restaurants. Most of meals that are offered in these restaurants are high in calories and fats. Studies found that peers have an influence on the behavior of overweight and obese children or those at risk of being overweight or obese because children pick up on the behaviors of their peers which could include lack of exercise, eating unhealthy foods, and not taking care of health and body image [14,15]. Further, the ability of children to achieve the recommended action, such as doing regular exercise instead of eating high- calorie foods, seems to be related to self-efficacy “beliefs of people in their ability to perform the recommended action” [16]. Consequently, the presence of delicious high calorie foods at home or school may not help children to make the correct decision and can lead to negative impacts, particularly on the children who have low self-efficacy. Although this chronic disease is multifactorial in origin, investigating related factors and working on reducing them in a systematic way will progress toward reducing the burden of disease. In addition, studies that estimated the prevalence of obesity among Jordanian adolescents and factors associated with it are lacking. Therefore, this study aimed at estimating the prevalence rate of overweight and obesity among adolescents in Irbid Governorate, North Jordan and in examining its relation to SES and individuals' perceptions on health.

## Methods

**Setting:** this was a cross sectional study conducted in Irbid City, which is Jordan's second largest city located about 85 km north of Amman, Jordan's capital. The population of the Governorate of Irbid is 1.77 million which represents about 17% of the total population of Jordan. Approximately, 38% of the Governorate of Irbid population is children less than 15 years old [17].

**Study population and sample:** all students in the age group 12 to 17 years in six participating schools were considered as the target population, giving a total of 2,803 students. In this study, 836 successfully completed the study survey, with a response rate of 96.1% (836 out of 870 students who were approached to participate in the study). After analyzing the raw data, the researcher excluded 99 students because they were underweight. Thirty-seven other students were excluded because they did not answer all the items of the questionnaire. The final number of students who completed the questionnaire and were included in the final analysis was seven hundred and one. The participating schools vary in their nature, type and region. It includes public versus private schools, girls versus boys, and urban versus rural areas. The study population was selected in multistage five steps sampling procedure: determining the number of schools in Irbid Governorate, selecting the schools purposefully taking into consideration the schools which have classes from the 7^th^-11^th^ grade, determining the number of classes in each grade, randomly selecting more than one class from each grade, randomly selecting half of the students from classes that were chosen randomly.

**Study instrument and pilot testing:** after reviewing the related literature, the researcher modified a version of a survey that was designed as an instrument published in 2008; entitled “Obesity and related factors among students grade 7-12 in Phuttha Monthon District, Nakhon Pathom Province, Thailand” [18]. The survey was translated from English to Arabic that was used in data collection of this study. For the purposes of the current study, an easy understood Arabic language questionnaire was distributed to the participating students. The study survey includes general information about the students' socio-demographic characteristics. Before starting the data collection process, a pilot test was carried out in one of public schools' classes. Some appropriate modifications were made mainly in editing. Some questions were joined in a box to separate them from others. Some of instructions were written and the type and size of font were changed to make the questions easier to read and understand.

**Measurements and data collection:** a consent sheet was distributed to students to ask their parents' approval. Research assistants were trained on how to take weight and height and to compute body mass index (BMI). BMI was calculated as body weight divided by height squared (kg/m²) and classified according to the Ministry of Health. The classification is indicated in the following (Table 1). The research team had to visit the schools more than one time to take the BMI measures of the whole sample since it was difficult to take them all at once.

**Table 1:** The classification of the body mass index

Classification	Body Mass Index
Underweight	Less than 18.5
Normal	From 18.5-24.9
Overweight	From 25-29.9
Obesity	Equal or more than 30

**Analysis:** Statistical Package for the Social Sciences (SPSS) Version 20 was used in all analyses. Analysis of the data included descriptive statistics for demographic information and perception about health. Associations of demographic information and perceptions about health with overweight/obesity was done through Chi-Square test of independence. All variables demonstrating a p-value of less than 0.05 were considered statistically significant.

**Ethical consideration:** all the aspects of the study were reviewed and approved by the Internal Review board at Jordan University of Science and Technology (Faculty of Medicine) and the Ministry of Education. Students were informed that the participation in this study was voluntary and they were assured of the confidentiality of all information obtained.

## Results

Descriptive statistics were used to report socio-demographic data. The whole study was conducted in the first directorate of Irbid in Jordan. The schools' categories consisted of 32.8% governmental schools and 67.2% private schools. The number of participants was 701, 53.6% were males and 46.4% were females. About 70% were 15 years or younger, with age ranging from 12 to18 years old. More than 73% of the fathers of the students had diploma or more, while almost 69% of mothers of the students had diploma or more. For monthly household income, the researcher found that 39.5% were having 600 JD or less with mean BMI of 23.89, about 36.1% were having 601 to 1200 JD with mean BMI of 22.72 and 24.4% were having 1201 JD or more with mean BMI of 24.65. Students with normal BMI had a mean BMI of 21.45 (+1.76). Those who were obese, or overweight were 202 (28.8%) students and had a BMI of 29.1 (+4.05). Table 2 illustrates measurement of students' BMI. The relationship of socioeconomic status and BMI is illustrated in Table 3. Mother's education and occupation, monthly household income, daily pocket income, and ranking between brothers and sisters were significantly associated with BMI among other SES factors. Study participants varied in their perceptions and practices of healthy habits related to weight. Their responses in relationship with BMI is shown in Table 4. Many of these perceptions or habits were significantly associated with BMI.

**Table 2:** Descriptive statistics of the BMI according to its specific classification

Body Mass Index (BMI)						
Number of Students	Prevalence%	Mean	Standard Deviation	Median	Minimum	Maximum
**Normal BMI**						
499	71.18	21.45	1.76	21.31	18.51	25.00
**Overweight and Obesity**						
202	28.82	29.10	4.05	27.66	25.08	44.30

SD: standard deviation; BMI: Body Mass Index

**Table 3:** The association of socioeconomic status with BMI of students

Socio-demographicVariables	Total		Body Mass Index		Mean	Std. Dev.	P-value
	#	%	Normal	Overweight and Obesity			
**School category**							
Private	471	67.2	127	344	23.53	4.54	0.121
Governmental	230	32.8	75	155	23.89	3.93	
**Residence place**							
Rural	140	20.0	36	104	23.48	3.95	0.365
Urban	561	80.0	166	395	23.69	4.45	
**Grade**							
7^th^	104	14.8	26	78	23.12	3.58	0.661
8^th^	142	20.3	38	104	23.30	4.35	
9^th^	182	26.0	51	131	23.77	4.36	
10^th^	163	23.3	53	110	23.83	4.83	
11^th^	110	15.7	34	76	24.14	4.23	
**Age**							
13 years and less	150	21.4	36	114	22.86	3.73	0.111
14 years	179	25.5	43	136	23.10	4.10	
15 years	154	22.0	54	100	24.26	4.63	
16 years	128	18.3	40	88	24.28	4.99	
17 years and more	90	12.8	29	61	24.13	4.04	
**Gender**							
Male	376	53.6	113	263	23.77	4.87	0.437
Female	325	46.4	89	236	23.52	3.65	
**Father's Education**							
High school and Less	184	26.2	63	121	24.21	4.20	0.059
Diploma and more	517	73.8	139	378	23.45	4.39	
**Father's Occupation**							
Don't Work	19	2.7	8	11	25.60	4.98	0.195
Work	682	97.3	194	488	23.60	4.32	
**Mother's Education**							
High school and Less	218	31.1	79	139	24.25	4.07	0.004
Diploma and more	483	68.9	123	360	23.38	4.45	
**Mother's Occupation**							
Work	230	32.8	52	178	23.17	4.25	0.011
Don't Work	471	67.2	150	321	23.89	4.38	
**Monthly Household Income**							
600 JD and less	277	39.5	89	188	23.89	4.34	0.013
601 to 1200	253	36.1	56	197	22.72	3.41	
1201 and more	171	24.4	57	114	24.65	5.26	
**How much your daily pocket income**							
0.5 JD and less	393	56.1	87	306	22.89	4.09	0.000
> 0.5 JD	308	43.9	115	193	24.62	4.48	
**Number of family members**							
4 and less	47	6.7	9	38	23.80	5.34	0.186
5 to 8	563	80.3	162	401	23.59	4.33	
9 and more	91	13.0	31	60	23.95	3.89	
**Your ranking between brother's and sister's**							
4 and less	562	80.2	149	413	23.50	4.37	0.020
5 to 8	126	18.0	47	79	24.19	4.25	
9 and more	13	1.9	6	7	24.90	4.02	

**Table 4:** Perceptions about health in relation to BMI of students

Perceptions Variables	Total		Body Mass Index		*χ*^2^	P-value
	#	%	Overweight and Obesity	Normal		
**How many family members are considered obese/overweight**						
None	255	36.4	44	211	**84.072**	< 0.001
1 member	235	33.5	49	186		
2 members	113	16.1	50	63		
at 3 and more	98	14.0	59	39		
**I feel that my weight**						
Normal and Less than normal weight	485	69.2	62	423	**206.605**	< 0.001
overweight	166	23.7	99	67		
Obese	50	7.1	41	9		
**Are you interested in your weight**						
Very interested	252	35.9	61	191	**14.514**	0.001
Little Interested	333	47.5	91	242		
Not interested	116	16.5	50	66		
**How much do think you have to eat at any meal?**						
Small amount	87	12.4	23	64	**20.972**	< 0.001
Suitable amount	489	69.8	122	367		
Large amount	125	17.8	57	68		
**Do you prefer a certain type of food at home?**						
Yes	553	78.9	153	400	1.685	0.194
No	148	21.1	49	99		
**Do you prefer eating food that contains vegetables?**						
Yes	510	72.8	128	382	**12.614**	< 0.001
No	191	27.2	74	117		
**Do you believe that your family has a role in improving your food habits?**						
Yes	552	78.7	158	394	0.047	0.828
No	149	21.3	44	105		
**During this school year, did you learn any lessons about the types of health food that must be eaten?**						
Yes	446	63.6	126	320	0.191	0.662
No	255	36.4	76	179		
**During this school year, did you learn any lessons about the harmful effect of eating too much junk food, especially the overweight or obese?**						
Yes	421	60.1	133	288	**3.958**	0.047
No	280	39.9	69	211		

## Discussion

Obesity is a growing public health problem of epidemic proportions in both developed and developing countries. Among children and adolescents, obesity is becoming a big concern in for its link with many future life-threatening conditions including atherosclerosis, diabetes mellitus and stroke. The present study provides better understanding of the factors related to adolescent obesity in Jordan. Our findings indicated that 19.4% of the surveyed school children were overweight while 5.6% of the children were obese. The study showed that there were no significant differences in BMI between males and females of the study population. This result is in contrast with the findings of Al Kloub *et al*. (2010) that surveyed school children 15-16 years in age, in which a higher frequency of obesity were found among males [19]. The differences in the gender-based distribution of obesity between the two studies could be a result of differences in the age range of the population of each respective study. Another study by Zhang *et al*. (2018) found that boys had higher odds for overweight or obesity than girls [13]. On the other hand, our findings are consistent with the results of Whitaker and Orzol (2006), which did not report a gender-based difference in the prevalence of obesity among school children [20]. Another important factor that was significantly associated with obesity among adolescents was the employment status of the mother. There was a significant difference between children of working mothers and non-working ones. These findings are consistent with several other reports from various geographical regions/ethnicities including studies in developed or developing countries; the uniformity of these findings could indicate a possible global trend [19,21,22]. Several reasons could explain this observation among Jordanian school children; firstly, maternal employment may be associated with a shorter time the mother or her partner spends on meal preparation. Accordingly, households of working mothers may more likely depend on restaurants for providing meals which tend to be of more calorie dense than meals prepared at home. Secondly, children of working mothers are more likely to spend time under the supervision of others, including teachers and peers at school, which might have a positive or negative impact in terms of children perceptions and behaviors [11]. However, the higher prevalence of obesity among children of working mothers were not consistent across all studies [23,24].

The main reason that influenced the status of children BMI was the parents' economic status; another predictor explored in this study. There was a positive association between income (total family or daily pocket) and being overweight/obese in our study. Findings suggest that family income may modify total calorie intake, dietary behaviour or physical activity of school children [25]. School children that are raised within families of higher total income have a wider range of food choices including food served at restaurants. Meals prepared and served at restaurants are usually more calorie dense and are higher in fat and sodium content. Moreover, a higher daily pocket income of the child could result in less strict control of children dietary behaviour by the parents; a factor that may lead to a higher consumption of calorie dense “fast” food [6,13,20]. Another determinant of childhood obesity in our report was the rank of the child in a big family size, with children belonging to larger families more likely of being overweight/obese. The exact reason of this finding warrants further investigation but could be related to children of larger families receiving less instruction from their parents regarding their food choices. Moreover, parents of fewer children regardless of the child's rank among siblings could be more educated and could spare more time to supervise their children. Accordingly, parents of smaller families may well be more attentive of their children's dietary behaviour and physical activity [12,25,26]. All these SES could affect the child’s perception about eating habits, physical activity and health in general, mainly affected by parental weight status, their views about health and obesity, and maternal education [27,28].

Perceptions of school-age children in this study were found to be a significant determinant of obesity/overweight prevalence. In this regard, perceptions of family members viewed as obese, perception and interest in own weight, meal amount, preference in vegetables in meals, and the harmful effect of eating junk food were significantly related to obesity/overweight prevalence. Similar results were reported by other studies [27,28]. These factors are related to SES and/or mother's education level. Nonetheless, a working mother who is educated or from a high SES might not have enough time to educate or direct her child toward healthy food, and thus, the child's perception towards eating habits will be affected by the social environment. Although our results highlight a relationship between SES and childhood obesity, the findings of this study still require further confirmation across several areas in Jordan. There is a wide range in the SES of families in Jordan and this study only collected information from school children of Northern Jordan. For example, families in the central region of Jordan, including the capital Amman, have a higher total family income; have smaller families and a higher percentage of working mothers. Accordingly, results from the central region of Jordan may be different from the results we obtained from Northern Jordan. Moreover, this study only reported data collected from school children from ages 12 to 17 and it is thus difficult to generalize the trends obtained from this data set to children of other ages. Indeed, it is well recognized that energy metabolism is highly affected by hormonal status which experiences a wide range of variation during childhood and early development. Finally, it should be recognized that this report collected data using a cross sectional design which is inherently impossible to use in order to establish true causal relationships between the different variables.

## Conclusion

According to the results of this study and those reported from similar ones, future research is warranted across different geographical regions of Jordan to further confirm our findings. In view of the relatively high prevalence rates of childhood obesity and their direct effect on childhood and future adulthood health, effective intervention strategies should be implemented in schools and other primary care settings. The above strategies should not only involve students and primary care providers, but also should include children parents and their families. These strategies should involve awareness programs of the role of dietary habits, physical activity and lifestyle in establishing and maintaining a healthy living. Moreover, they should emphasize the importance of establishing an open communication channel between children and their parents to better supervise children and influence their food choices.

### What is known about this topic

- Previous studies in this region and Jordan have tackled the issue of obesity determinants among children with other objectives;
- Obesity among children were explored in the school-age 6-12.

### What this study adds

- Obesity among children were explored in the school-age 13-17;
- The effect of perceptions of children about the meals and obesity;
- The effect of the money allowance the child has in school.

## Competing interests

The authors declare no competing interests.
